# The Heart of the World

**DOI:** 10.5334/gh.1288

**Published:** 2024-01-25

**Authors:** Mariachiara Di Cesare, Pablo Perel, Sean Taylor, Chodziwadziwa Kabudula, Honor Bixby, Thomas A. Gaziano, Diana Vaca McGhie, Jeremiah Mwangi, Borjana Pervan, Jagat Narula, Daniel Pineiro, Fausto J. Pinto

**Affiliations:** 1 Institute of Public Health and Wellbeing, University of Essex, Colchester, UK; 2 Department of Non-Communicable Disease Epidemiology, London School of Hygiene & Tropical Medicine, London, UK; 3 World Heart Federation, Geneva, Switzerland; 4 MRC/Wits Rural Public Health and Health Transitions Research Unit (Agincourt), School of Public Health, Faculty of Health Sciences, University of the Witwatersrand, Johannesburg, South Africa; 5 Brigham and Women’s Hospital, Cardiovascular Medicine, Boston, USA; 6 Harvard Medical School, Boston, USA; 7 American Heart Association, Dallas, USA; 8 McGovern Medical School at UTHealth, Houston, USA; 9 Department of Medicine, University of Buenos Aires, Buenos Aires, Argentina; 10 Santa Maria University Hospital, CAML, CCUL, Faculdade de Medicina da Universidade de Lisboa, Lisbon, Portugal

**Keywords:** Cardiovascular health, CVDs risk factors, WHF policy index, CVD global data, WHF observatory

## Abstract

Cardiovascular diseases (CVDs) are the leading cause of mortality globally. Of the 20.5 million CVD-related deaths in 2021, approximately 80% occurred in low- and middle-income countries.

Using data from the Global Burden of Disease Study, NCD Risk Factor Collaboration, NCD Countdown initiative, WHO Global Health Observatory, and WHO Global Health Expenditure database, we present the burden of CVDs, associated risk factors, their association with national health expenditures, and an index of critical policy implementation.

The Central Europe, Eastern Europe, and Central Asia region face the highest levels of CVD mortality globally. Although CVD mortality levels are generally lower in women than men, this is not true in almost 30% of countries in the North Africa and Middle East and Sub-Saharan regions. Raised blood pressure remains the leading global CVD risk factor, contributing to 10.8 million deaths in 2019. The regions with the highest proportion of countries achieving the maximum score for the WHF Policy Index were South Asia, Central Europe, Eastern Europe, and Central Asia, and the High-Income regions. The Sub-Saharan Africa region had the highest proportion of countries scoring two or less.

Policymakers must assess their country’s risk factor profile to craft effective strategies for CVD prevention and management. Fundamental strategies such as the implementation of National Tobacco Control Programmes, ensuring the availability of CVD medications, and establishing specialised units within health ministries to tackle non-communicable diseases should be embraced in all countries. Adequate healthcare system funding is equally vital, ensuring reasonable access to care for all communities.

## Introduction

Cardiovascular diseases (CVDs) are the leading cause of mortality globally, responsible for a significant number of deaths and disabilities. In 2021 alone, CVDs accounted for 20.5 million deaths, comprising approximately one-third of all global deaths [1]. While cardiovascular conditions were traditionally considered *diseases of affluence*, this is no longer the case. Over three-quarters of CVD-related deaths occur in low- and middle-income countries (LMICs) [2].

Moreover, these deaths are the primary contributor to premature non-communicable disease (NCD) mortality. Ischemic heart disease, specifically, stands as the leading cause of premature death in 146 countries for men and 98 countries for women [3]. The complexity of the global picture is exacerbated by the levels of inequalities in the impact of CVDs. Notably, LMICs experience higher rates of premature mortality from CVDs compared to high-income countries (HICs), while the reduction in age-standardised mortality rates is progressing more slowly in LMICs.

In May 2012 the World Health Assembly adopted the target of a 25% reduction in premature mortality from noncommunicable diseases by 2025. In May 2013 the NCD Global Monitoring Framework was launched during the World Health Assembly. Its primary objectives were to drive progress in the prevention and control of NCDs, serve as a foundation for advocacy, raise awareness, strengthen political commitment, and promote global action against NCDs. The framework encompassed nine voluntary global targets focused on behavioural and metabolic risk factors, as well as national healthcare system responses [4].

Over a decade has passed since international organisations and governments have been working towards achieving these global targets and reducing premature mortality. However, the current pace of decline is insufficient. Based on trends observed between 2010 and 2016, it is estimated that less than 20% of countries will achieve the UN Sustainable Development Goal Target 3.4 (SDG 3.4) – a one-third reduction in premature mortality from NCDs by 2030 [5]. Most countries, particularly those classified as low- or lower-middle income, need to accelerate progress to meet these targets.

This paper summarises the World Heart Report 2023,^1^ which is a comprehensive overview of the trends and current landscape of the key dimensions of cardiovascular health. By synthesising epidemiological, policy, and economic data, the World Heart Report provides information that can help guide policymakers at national and international levels in identifying and addressing the most critical priorities to address cardiovascular conditions.

## Methods and data

The analysis of the burden of cardiovascular diseases and associated risk in this study utilised data from global initiatives including the Global Burden of Disease Study [6], the NCD Risk Factor Collaboration [7], and the NCD Countdown 2030 initiative [3]. These have been extensively described elsewhere and are considered the most valid and reliable sources for global cardiovascular data. In selecting data sources, priority was given to those that offered a high level of disaggregation (by sex, age, country, region), data completeness, richness of the raw data informing the model, and comparability of estimates.

Absolute numbers of death, age-standardised death rates, and the probability of dying between 30 and 70 years of age^2^ have been reported to describe the burden of cardiovascular mortality. Analysis of risk factors focused on nine specific factors. These factors were selected based on the six risk factors outlined in the WHO NCD Global Monitoring Framework, along with two additional risk factors—non-HDL cholesterol levels and air pollution. The inclusion of these two additional factors was based on their substantial impact on the prevalence and mortality rates of CVDs (Table 1).

**Table 1:** Mortality and risk factors indicators, source, and year.


INDICATOR	SOURCE	YEAR

**Mortality**		

Ischemic heart disease	Global burden of disease	1990–2019

Stroke	Global burden of disease	1990–2019

All other CVDs	Global burden of disease	1990–2019

**Risk factors**		

Diabetes	NCD risk factor collaboration	1975–2014

Raised blood pressure	NCD risk factor collaboration	1975–2015

Obesity	NCD risk factor collaboration	1975–2016

Lipids	NCD risk factor collaboration	1975–2018

Physical activity	Global burden of disease	1990–2019

Sodium intake	Global burden of disease	1990–2019

Alcohol consumption	Global burden of disease	1990–2019

Tobacco smoking	Global burden of disease	1990–2019

Ambient air pollution	Global burden of disease	1990–2019

Premature mortality (stroke)	NCD Countdown 2030	2015


To assess the risk factors’ country profiles, each country was ranked based on the level of the specific risk factor, ranging from the highest level to the lowest level. Subsequently, quintiles were generated for each risk factor, dividing the countries into five equal groups based on their respective levels of the risk factor.

To assess the association between CVD burden and health expenditure, additional data on Gross Domestic Product (GDP), Current Health Expenditure as a percentage of GDP (CHE), and Out of Pocket Expenditure (OOPS) were obtained from the WHO Global Health Expenditure database [8].

The *World Heart Federation Policy Index* (WHF Policy Index) provides an overview of national governments’ level of implementation of eight critical policies related to CVD health (Table 2). The expert panel of the WHF Advocacy Committee carefully selected these policy indicators as the most relevant for ensuring a healthy heart profile in populations. Data for the WHF Policy Index were obtained from the Global Health Observatory [9]. Each policy was assigned a score of 0 if it was not in place in the country and 1 if it was in place. The overall index score for each country ranged from 0 (no policies in place) to 8 (all policies in place). Complete information was available for 166 countries.

**Table 2:** Policy indicators, source, and year.


INDICATOR	SOURCE	YEAR

National tobacco control programmes	WHO Global Health Observatory	2018

Policy/strategy/action plan for CVD	WHO Global Health Observatory	2021

Operational Unit, Branch, or Dept. in Ministry of Health with responsibility for NCDs	WHO Global Health Observatory	2021

Guidelines/protocols/standards for the management of cardiovascular diseases	WHO Global Health Observatory	2021

Policy/strategy/action plan to reduce physical inactivity	WHO Global Health Observatory	2021

Policy/strategy/action plan to reduce unhealthy diet related to NCDs	WHO Global Health Observatory	2021

Policy/strategy/action plan to reduce the harmful use of alcohol	WHO Global Health Observatory	2021

Availability of ACE inhibitors, Aspirin (100 mg) and Beta blockers in the public health sector	WHO Global Health Observatory	2021


*Note*: Availability of ACE inhibitors, Aspirin (100 mg) and Beta blockers in the public health sector is a combination of the three indicators 1) availability of ACE inhibitors in the public health sector; 2) availability of Aspirin (100 mg) in the public health sector; 3) availability of Beta blockers in the public health sector. If all 3 available the overall score was set to 1 if one or more not available the overall score was set to 0.

Trends and levels are presented for both regions and individual countries. Countries were classified into regions based on the Global Burden of Diseases classification, which includes Central Europe, Eastern Europe, Central Asia, and the High-Income region as distinct regions (https://www.healthdata.org/sites/default/files/files/Projects/GBD/GBDRegions_countries.pdf). This division into more regions allows for more detailed comparisons of mortality and risk factor rates across significant geographical areas. To assess the association between CVD burden and health expenditure indicators the Pearson’s correlation coefficient was used.

## Results

**Figure 1:**
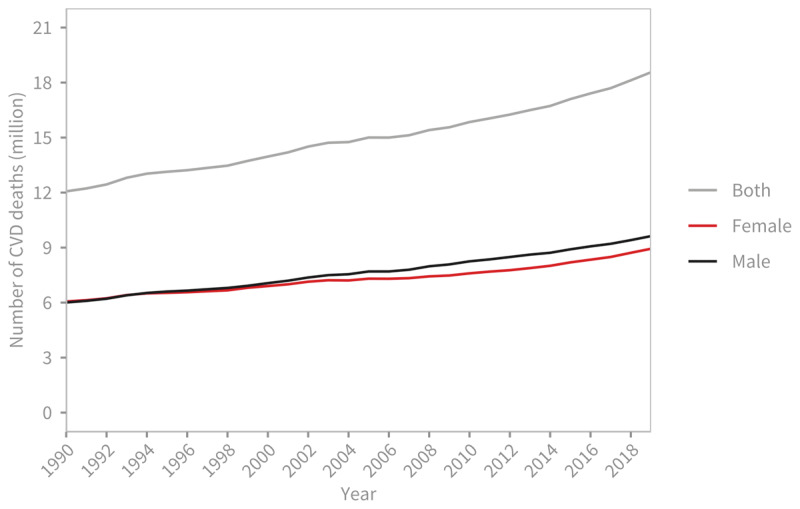
Global trends in number of deaths due to cardiovascular diseases, 1990–2019. Source: Institute for Health Metrics and Evaluation (IHME). GBD Compare Data Visualization. Seattle, WA: IHME, University of Washington, 2020. Available from http://vizhub.healthdata.org/gbd-compare.

**Figure 2:**
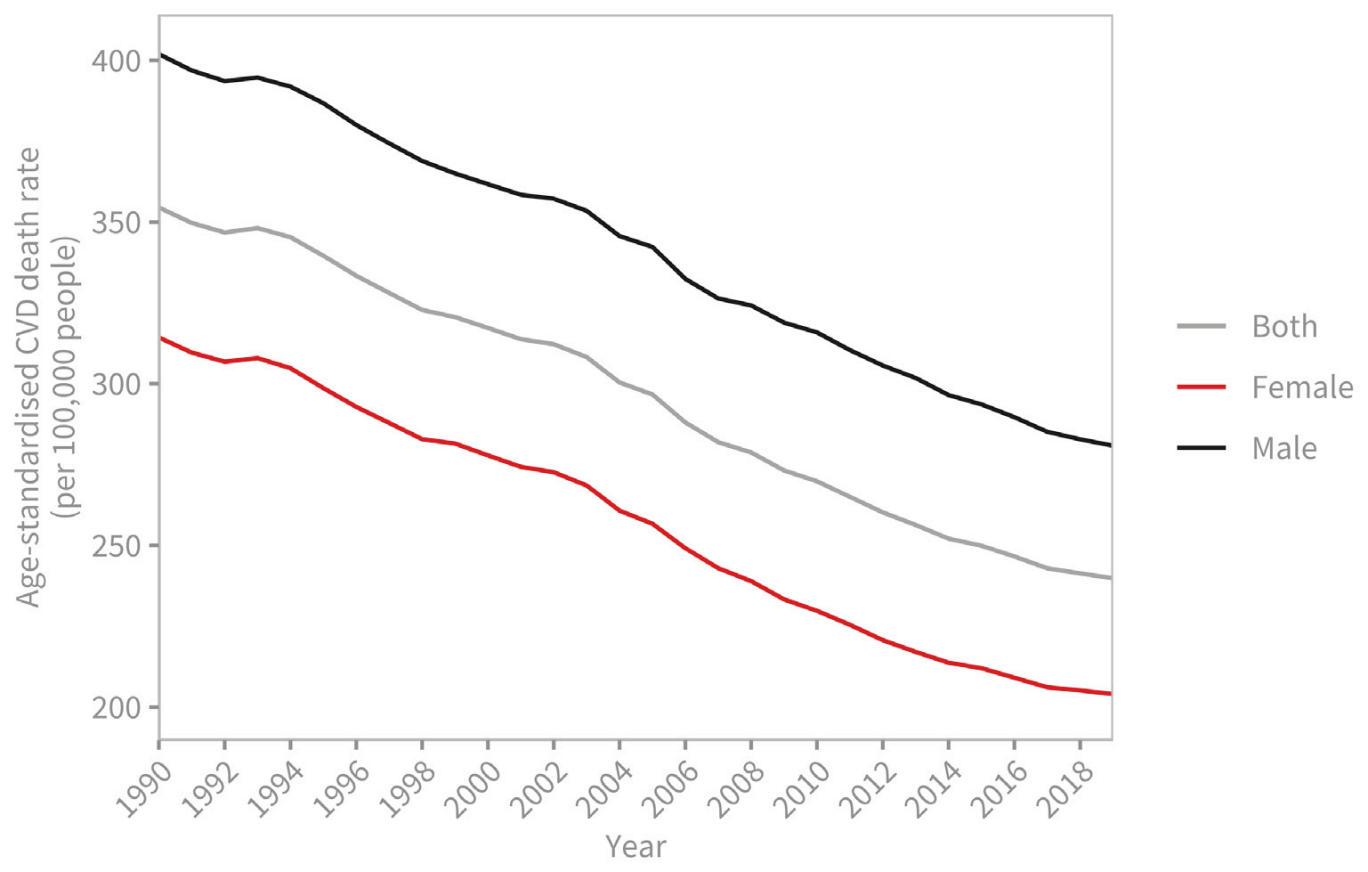
Global trends in age-standardised cardiovascular disease death rate (per 100,000 people), 1990–2019. Source: Institute for Health Metrics and Evaluation (IHME). GBD Compare Data Visualization. Seattle, WA: IHME, University of Washington, 2020. Available from http://vizhub.healthdata.org/gbd-compare.

The number of deaths due to acronym introduced in the introduction CVDs has increased over the years. In 1990, the estimated deaths stood at around 12.1 million [95% Uncertainty Interval: 11.4–12.6 million], equally distributed between men and women. By 2019, this number had risen to 18.6 million [17.1–19.7], with 9.6 million [8.9–10.3] deaths among men and 8.9 million [7.9–9.7] deaths among women (Figure 1). Overall, CVDs accounted for 33% of all global deaths in 2019, with ischemic heart disease (9.1 million deaths) and stroke (6.6 million deaths) contributing to 85% of CVD-related deaths. While the number of deaths due to CVDs over the last 30 years has increased, mostly due to the ageing and growth of the population, the age-standardised death rate, which accounts for changes in population demographics, has declined from 354.5 per 100,000 people [330.6–369.5] in 1990 to 239.9 per 100,000 people [219.4–254.9] in 2019 (Figure 2).

**Figure 3:**
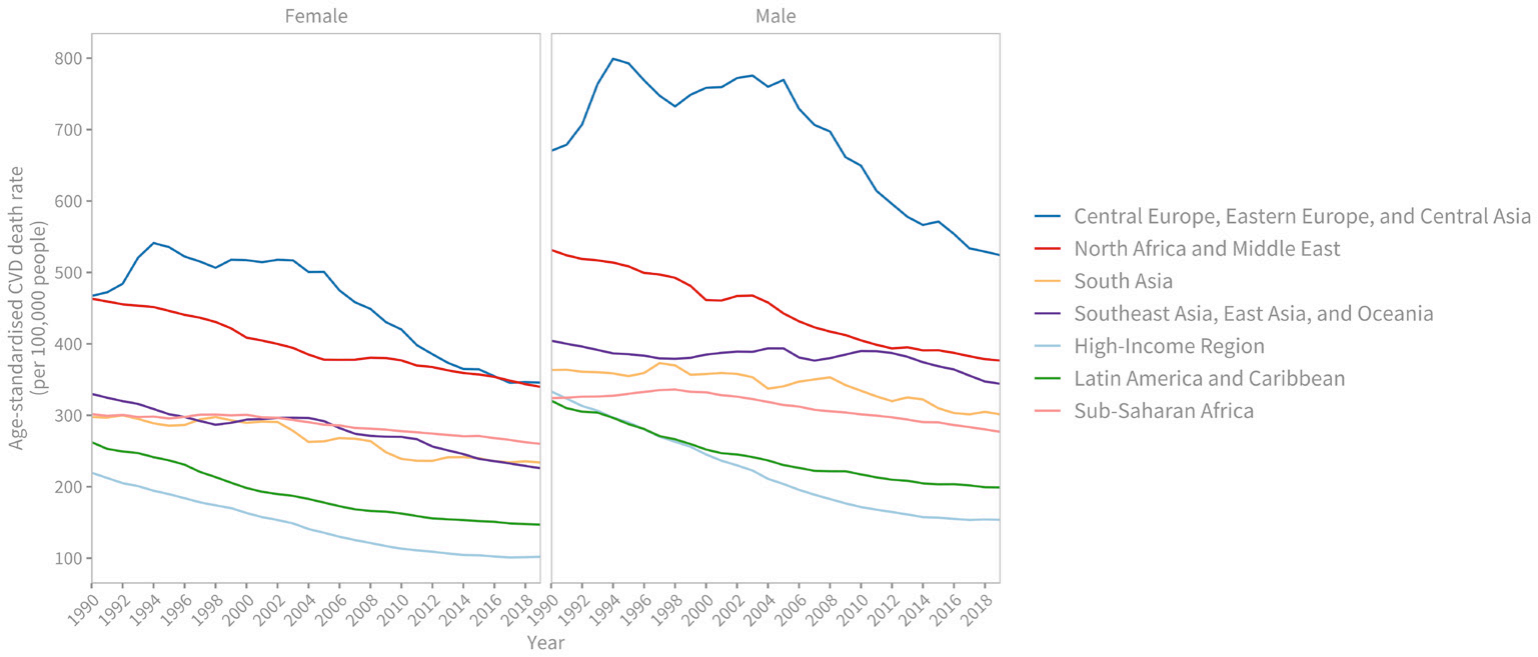
Regional trends in age-standardised cardiovascular disease death rate (per 100,000 people), 1990–2019. Source: Institute for Health Metrics and Evaluation (IHME). GBD Compare Data Visualization. Seattle, WA: IHME, University of Washington, 2020. Available from http://vizhub.healthdata.org/gbd-compare.

When examining regional trends, significant variations in the rate of decline in age-standardised CVD death rates become apparent (Figure 3). Between 1990 and 2019, all regions experienced a decline in age-standardised CVD death rates, but there are signs of this decline slowing down in the past decade. The High-income region exhibited the fastest average rate of decline for both men and women, with an average annual rate of change of 2.6%. On the other hand, the South Asia, the Southeast Asia, East Asia and Oceania, and the Sub-Saharan Africa regions had the slowest rates of decline. Notably, there was almost no improvement in CVD death rates for men in these regions.

In 1990, male age-standardised CVD death rates in the Sub-Saharan Africa region were 1.2 times higher than those observed in the High-income region. However, by 2019, the age-standardised rates in the Sub-Saharan Africa region were 2.1 times higher than those in the High-income region. For women in 2019, only the Latin America and the Caribbean region achieved CVDs death rates levels comparable to those observed in the High-income region in 1990, almost three decades before.

The Central Europe, Eastern Europe, and Central Asia region had the highest age-standardised death rates for males and females in both 1990 (670.2 [644.4–683.3] and 467.2 [436.4–481.7] deaths per 100,000 people respectively) and 2019 (524.1 [475.4–566.9] and 345.7 [308.1–376.3] deaths per 100,000 people respectively).

The North Africa and Middle East region had the second highest death rates for males (376.7 per 100,000 people [336.5–414.7] and females (339.8 per 100,000 people [298.9–374.2]) in 2019.

Ischemic heart disease stands as the leading cause of CVD mortality across all regions, except for women in Sub-Saharan Africa. Notably, in 2019, the death rate for cardiomyopathy and myocarditis among males in Central Europe, Eastern Europe, and Central Asia was more than three times higher (28.5 per 100,000 people [20.3–32.9]) than in the second highest region (Sub-Saharan Africa, 8.4 per 100,000 people [5.8–10.6]) and thirty times higher than the region with the lowest rates (South Asia, 0.8 per 100,000 people [0.6–1.1]).

### Cardiovascular deaths by sex

**Figure 4:**
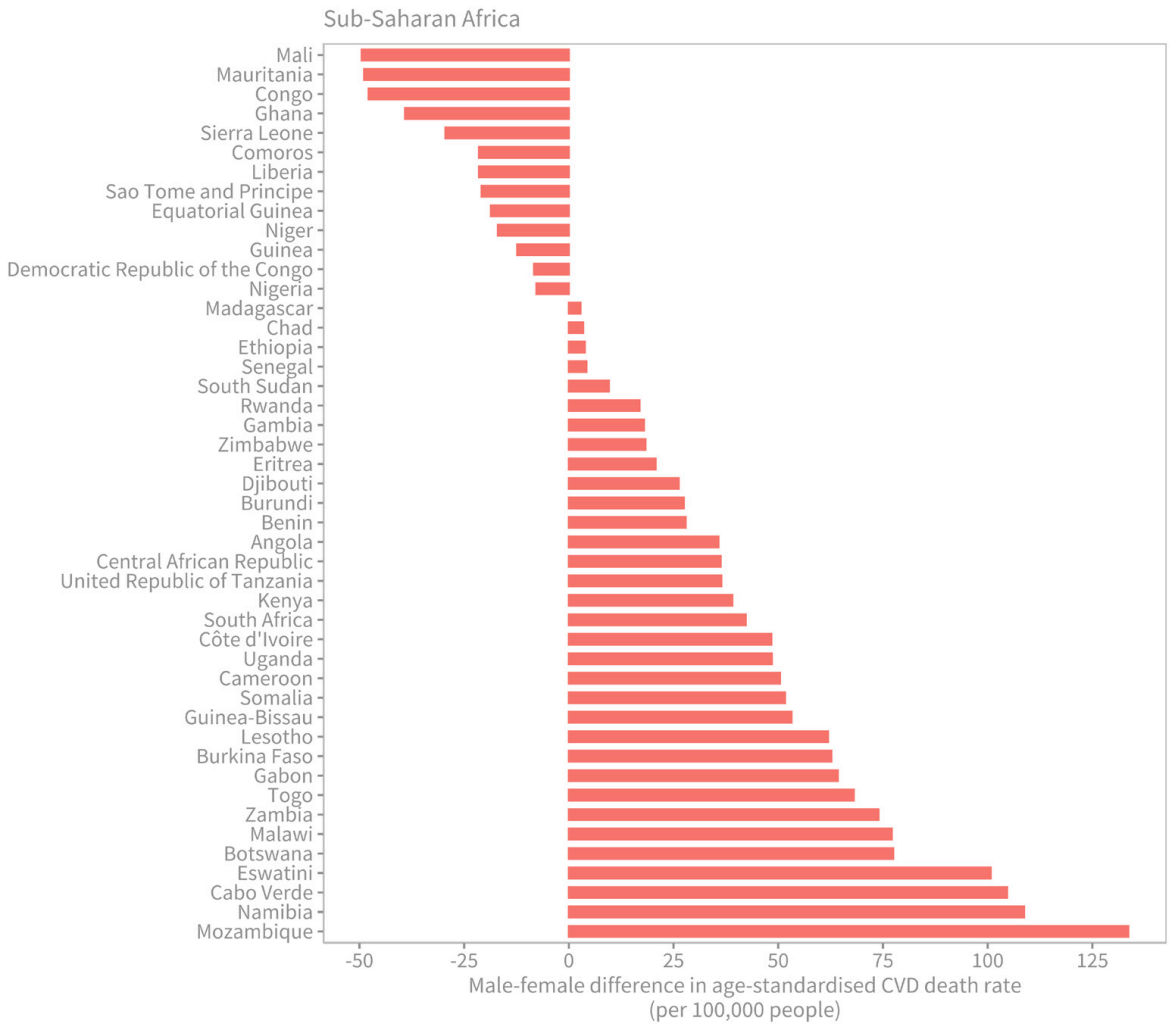
Male-female difference in age-standardised cardiovascular disease death rate (per 100,000 people) in sub-Saharan Africa by country, 2019. Source: Institute for Health Metrics and Evaluation (IHME). GBD Compare Data Visualization. Seattle, WA: IHME, University of Washington, 2020. Available from http://vizhub.healthdata.org/gbd-compare.

On a global scale, men exhibit higher age-standardised death rates compared to women. In 2019, this trend persisted across all countries in the High-income region, the Central Europe, Eastern Europe, and Central Asia region, as well as the South Asia region. However, in almost one-third of countries (six out of 21) in the North Africa and Middle East region, women experienced higher death rates from CVDs compared to men. Notable disparities were observed in Qatar (464.6 deaths per 100,000 people [395.0–536.5] for women versus 301.9 [241.4–368.4] for men), Egypt (600.0 [463.8–720.2] versus 491.6 [386.3–615.1] per 100,000 people), and Algeria (447.7 [384.1–509.5] versus 371.5 [306.9–449.0] per 100,000 people). Similarly, in 13 out of the 46 countries in the Sub-Saharan Africa region, women had higher CVDs death rates than men. The largest gaps were observed in Mali, Mauritania, Congo (Congo-Brazzaville), Ghana, and Sierra Leone (Figure 4). Two other countries where women experienced higher death rates than men were Haiti (475.3 [352.3–635.4] versus 419.8 [320.1–562.4] per 100,000 people) and Tokelau (388.7 [308.9–484.8] versus 312.2 [261.6–380.4] per 100,000 people).

### CVD premature mortality

**Figure 5:**
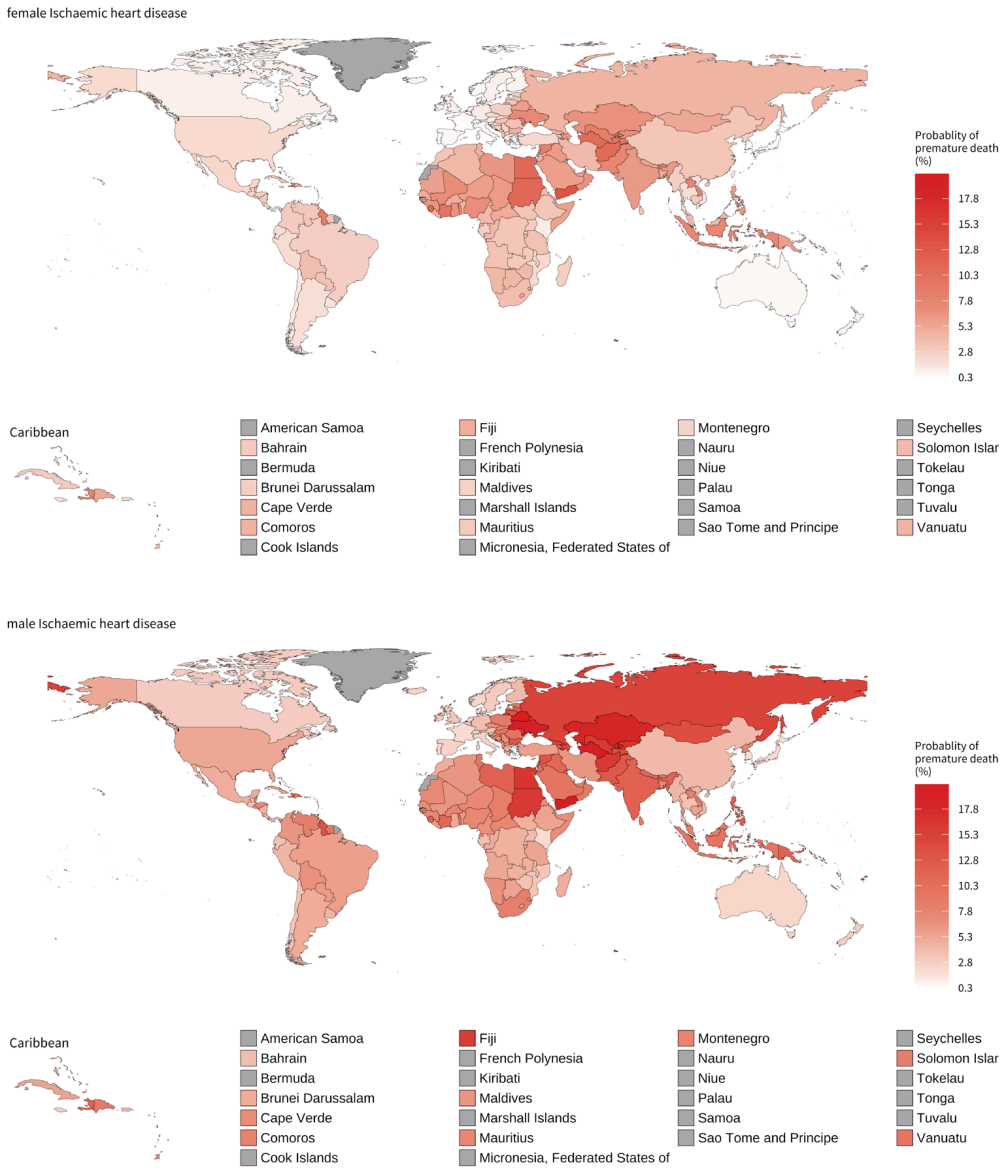
Probability of dying (reported as a percentage) in 2015 between 30 years and 70 years of age from ischaemic heart disease by sex. *Note*: Grey colour is used when no estimates were available (missing). Source: NCD Countdown [3].

Ischaemic heart disease emerges as the leading cause of premature death among all NCDs in most countries, impacting both men and women. The highest risk of premature mortality from ischaemic heart disease is observed in countries within the Central Europe, Eastern Europe, and Central Asia region, followed by the North Africa and Middle East region. The risk of premature mortality from ischaemic stroke among men is also highest in the Central Europe, Eastern Europe, and Central Asia region. Yemen stands out as the country with the highest risk of premature mortality across all countries, with men and women facing risks of 20.0% and 13.3%, respectively (Figure 5). The risk of premature mortality due to haemorrhagic stroke is particularly high in Mongolia (10.3% among men and 6.4% among women) and Turkmenistan (9.6% among men). Similarly, for ischaemic stroke, the highest risk (around 3%) among women is observed in Sub-Saharan Africa, and specifically in Western African countries, such as Ghana, Sierra Leone, and Côte d’Ivoire. In Sub-Saharan Africa, the risk of premature mortality from other cardiovascular diseases also tends to be highest among women.

### Risk factors

**Figure 6:**
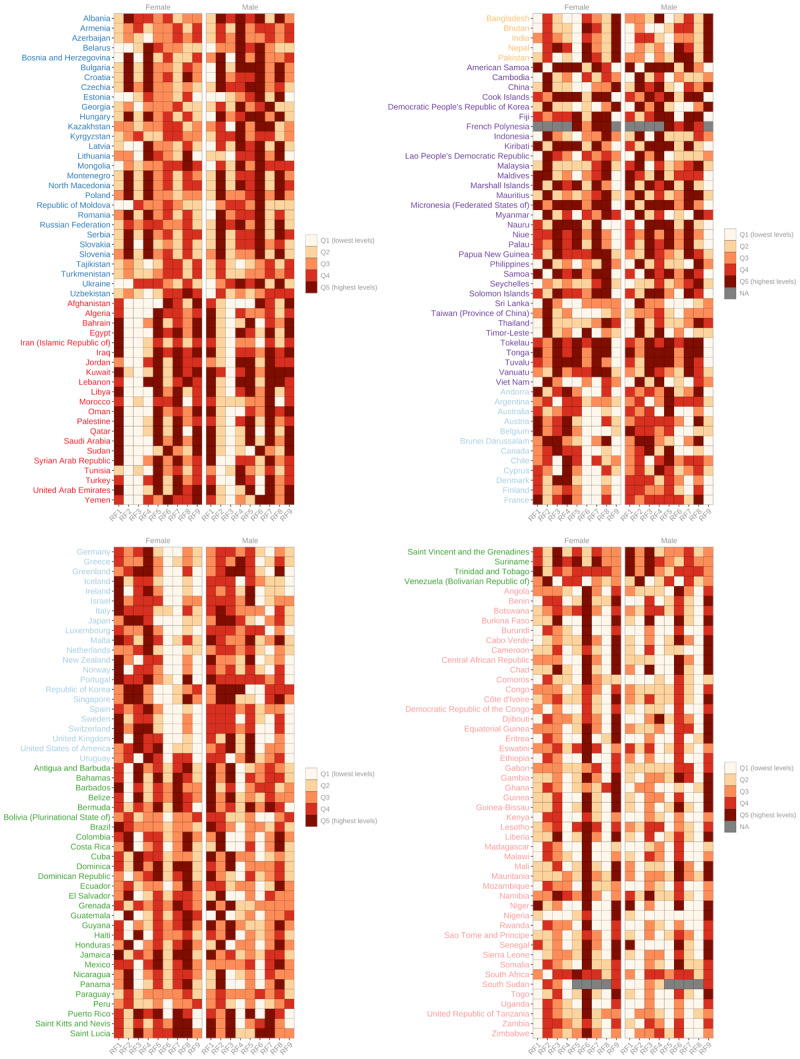
Comparative levels of risk factors by country and region. *Note (1)*: RF1 – Physical activity; RF2 – Sodium intake; RF3 – Alcohol consumption; RF4 – Tobacco smoking; RF5 – Obesity; RF6 – Raised Blood Pressure; RF7 – Diabetes; RF8 – Lipids; RF9 – Ambient air pollution. *Note (2)*: The figures display the global quintile into which each country falls for each risk factor. Source: See Table 1.

The risk factor profile provides a comprehensive understanding of the burden of CVD risk factors within and between countries (Figure 6). The analysis reveals notable variations across regions. In Central Europe, Eastern Europe, and Central Asia most countries exhibit high levels of sodium intake, raised blood pressure, and non-HDL cholesterol among men. Additionally, high levels of sodium intake and tobacco smoking are prevalent among women. Comparatively, countries in Central and Eastern Europe tend to have higher overall risk factor levels than those in Central Asia. Most countries in the High-income region are characterised by elevated levels of behavioural risk factors, including high sodium and alcohol consumption, tobacco smoking, and low physical activity. Both sexes also experience high levels of non-HDL cholesterol, while men have high obesity levels. The Latin America and Caribbean region shows greater levels of heterogeneity. However, low physical activity emerges as a significant risk factor across many countries. Generally, the Caribbean countries tend to have higher risk factor levels compared to other countries in the region. The North Africa and Middle East region is characterised by high levels of metabolic risk factors, particularly diabetes and obesity prevalence. Air pollution and physical inactivity are also notable risk factors. Middle Eastern countries generally exhibit higher risk factor levels compared to those in North Africa. All countries in South Asia face high levels of air pollution. Sodium intake, raised blood pressure, and diabetes are also prominent risk factors in South Asia for both sexes.

Countries in Oceania have high levels of diabetes, obesity, tobacco smoking, and low physical activity. Women are characterised by some of the highest levels of non-HDL cholesterol. In Southeast Asia and East Asia, high levels of sodium intake and non-HDL cholesterol are observed across all countries.

The Sub-Saharan Africa region is characterised by high levels of raised blood pressure in most countries. Air pollution levels are generally high, particularly in the Sahel countries. Southern Sub-Saharan African countries tend to have the highest levels of CVD risk factors within the region.

### Relationship between CVD mortality and health expenditure

The World Health Organization (WHO) and various civil society organisations have long advocated for countries to allocate a minimum of 5% of their Gross Domestic Product (GDP) towards healthcare. However, there are significant variations in the extent to which countries in each region achieve this target (Table 3). For instance, in South Asia, no countries meet the 5% target, with Bangladesh allocating 2.6% of its GDP to health expenditure, Bhutan allocating 3.6%, India allocating 2.9%, Nepal allocating 4.4%, and Pakistan allocating 2.8%.

**Table 3:** Proportion of countries spending at least 5% of GDP on health.


REGION	%

High-income	97

Central Europe, Eastern Europe, and Central Asia	85

Latin America and Caribbean	71

North Africa and Middle East	53

Southeast Asia, East Asia, and Oceania	50

Sub-Saharan Africa	45

South Asia	0


**Figure 7:**
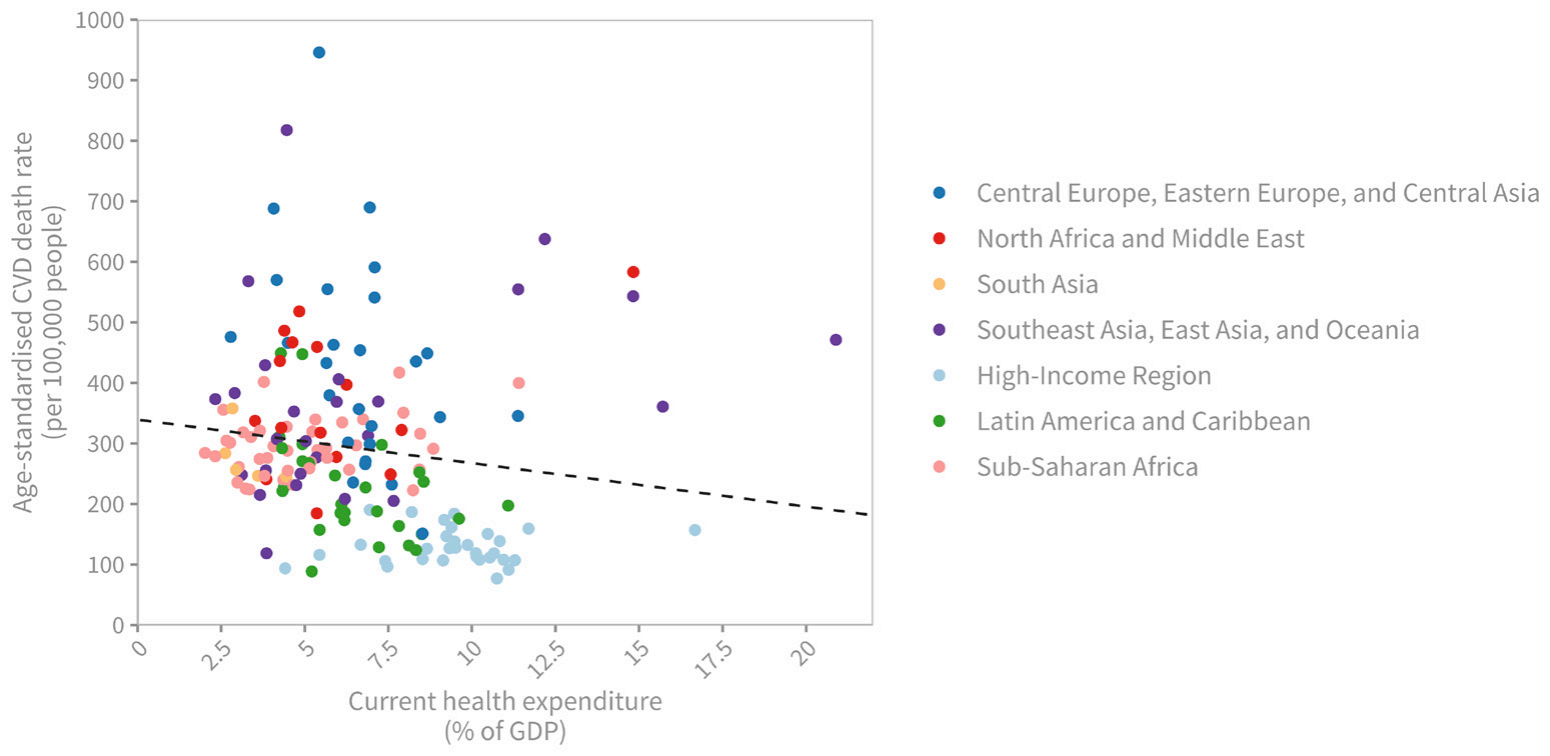
Age-standardised cardiovascular disease death rate (per 100,000 people), 2019, by current health expenditure (CHE) as share of national income (GDP). *Note*: Each dot represents a country, coloured by region. The dashed line shows the negative linear association between CVD death rates and national health expenditure (Pearson’s correlation coefficient = –0.15, p = 0.052). Sources: Institute for Health Metrics and Evaluation (IHME). GBD Compare Data Visualization. Seattle, WA: IHME, University of Washington, 2020. Available from http://vizhub.healthdata.org/gbd-compare; World Health Organization, Global Health Expenditure Database. Available from https://apps.who.int/nha/database/Select/Indicators/en.

**Figure 8:**
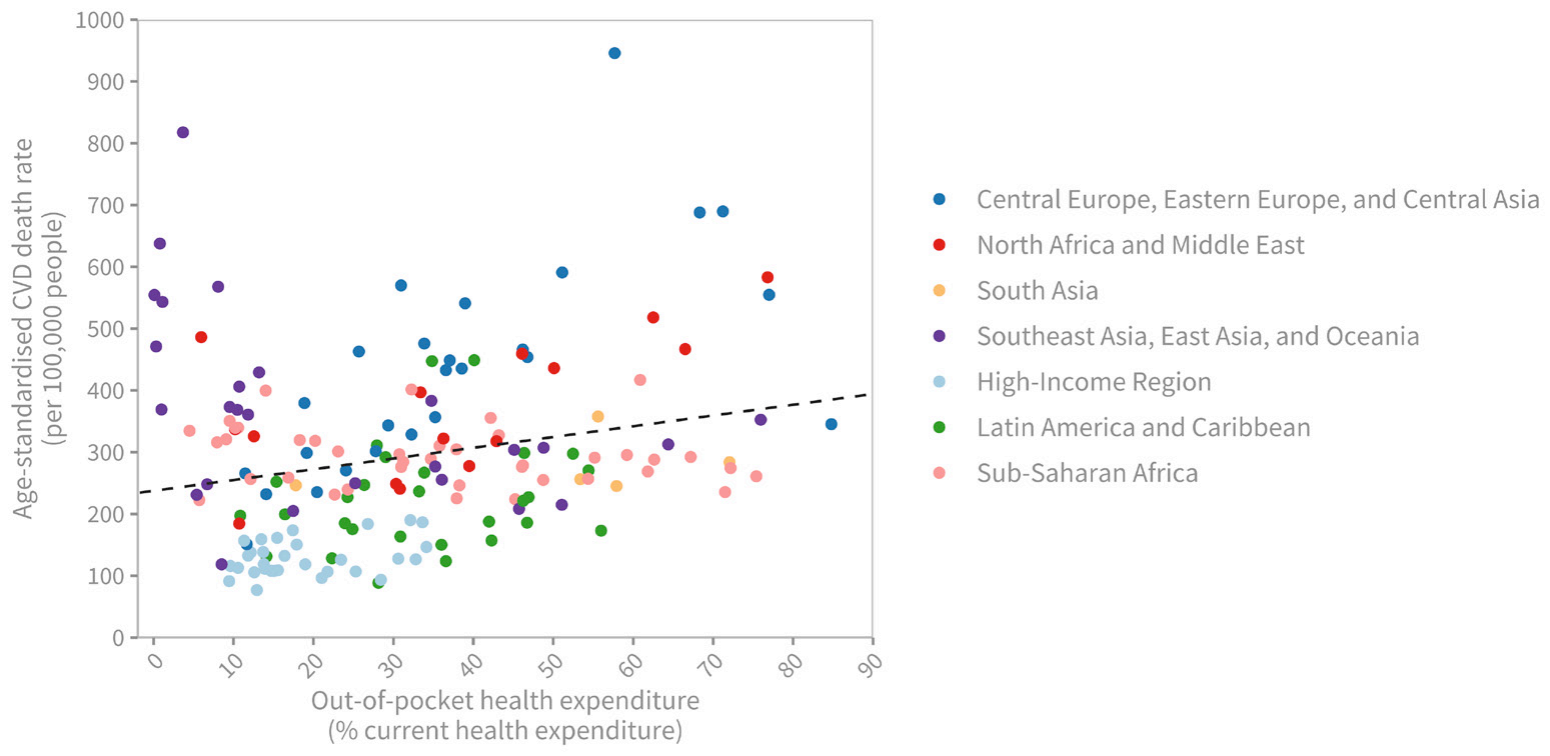
Age-standardised cardiovascular disease death rate (per 100,000 people), 2019, by out-of-pocket (OOP) health expenditure as share of current health expenditure (CHE). *Note*: Each dot represents a country, coloured by region. The dashed line shows a positive association between CVD death rates and OOP health expenditure as a share of national health expenditure (Pearson’s correlation coefficient = 0.23, p = 0.002). Sources: Institute for Health Metrics and Evaluation (IHME). GBD Compare Data Visualization. Seattle, WA: IHME, University of Washington, 2020. Available from http://vizhub.healthdata.org/gbd-compare; World Health Organization, Global Health Expenditure Database. Available from https://apps.who.int/nha/database/Select/Indicators/en.

There exists a correlation between the percentage of current health expenditure (CHE) relative to GDP and CVD death rates. Countries with lower CHE as a percentage of GDP tend to experience higher age-standardised CVD mortality rates (Figure 7). Additionally, a higher percentage of out-of-pocket expenditure (OOPS) relative to CHE is also associated with higher age-standardised CVD mortality (Figure 8).

### WHF policy index

**Figure 9:**
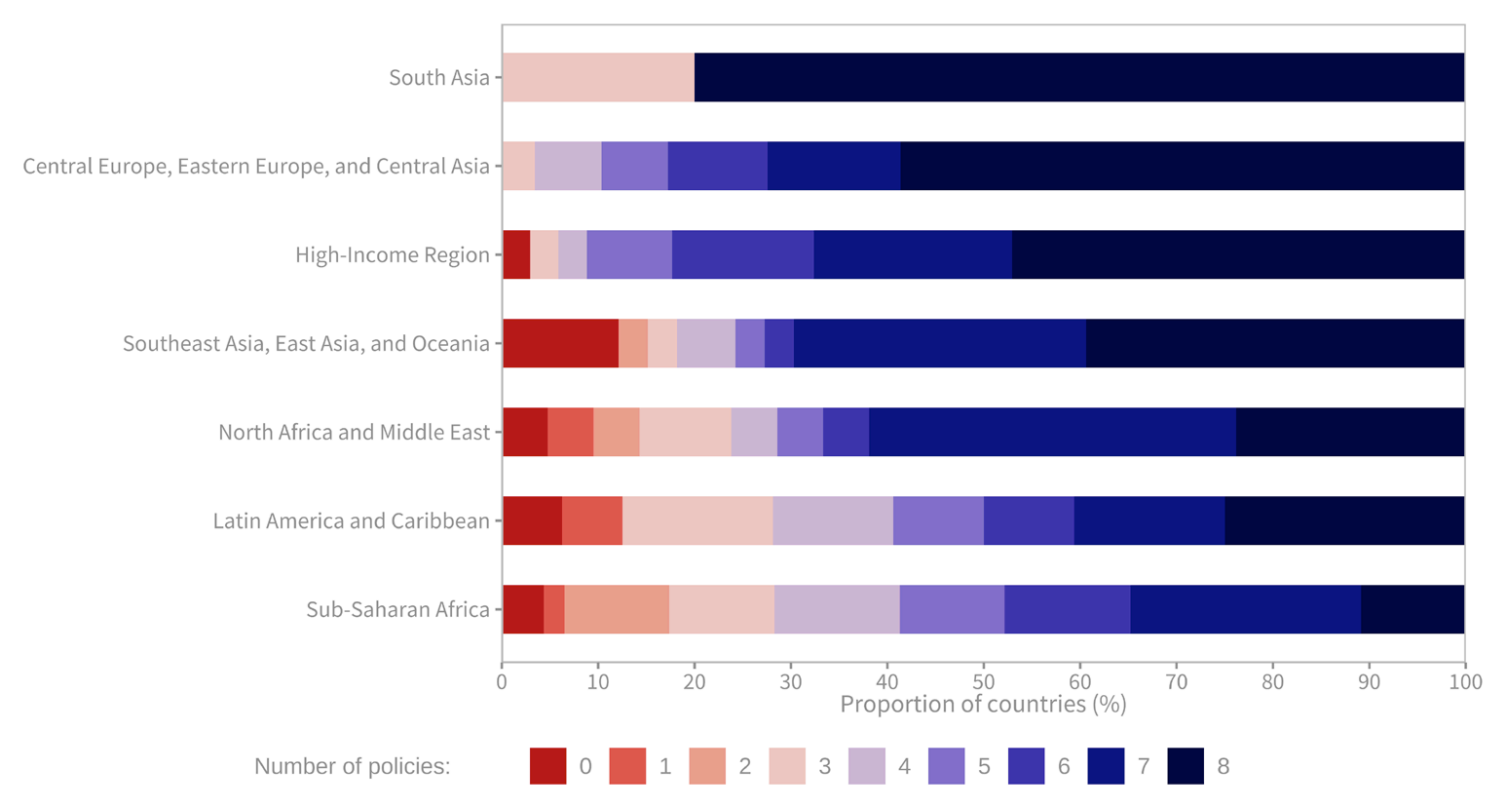
Number of key policies implemented to address cardiovascular diseases, by region (% of countries). Sources: Global Health Observatory. Available from: https://www.who.int/data/gho.

Globally, a significant majority of countries, 106 out of 166 countries with available information (64%), have at least seven out of the eight policies evaluated in place. The regions with the highest proportion of countries achieving the maximum score of eight were South Asia (80%), Central Europe, Eastern Europe, and Central Asia (68%), and the High-Income region (62%). In contrast, the Sub-Saharan Africa region had the lowest proportion of countries (13%) achieving the maximum score, and the highest proportion (16%) of countries scoring two or less. The Latin America and Caribbean region, as well as the Southeast Asia, East Asia, and Oceania region, also had countries with scores of one or two (Figure 9).

At a global level, the policy most commonly in place was the existence of national tobacco control programmes, which was the case in 91% of countries. This was followed by the existence of guidelines/protocols/standards for the management of CVDs (86%) and the existence of policy/strategy/action plans to reduce unhealthy diets related to NCDs (85%). The policy least frequently in place was the existence of an action plan to reduce the harmful use of alcohol, which was in place in 70% of countries.

**Figure 10:**
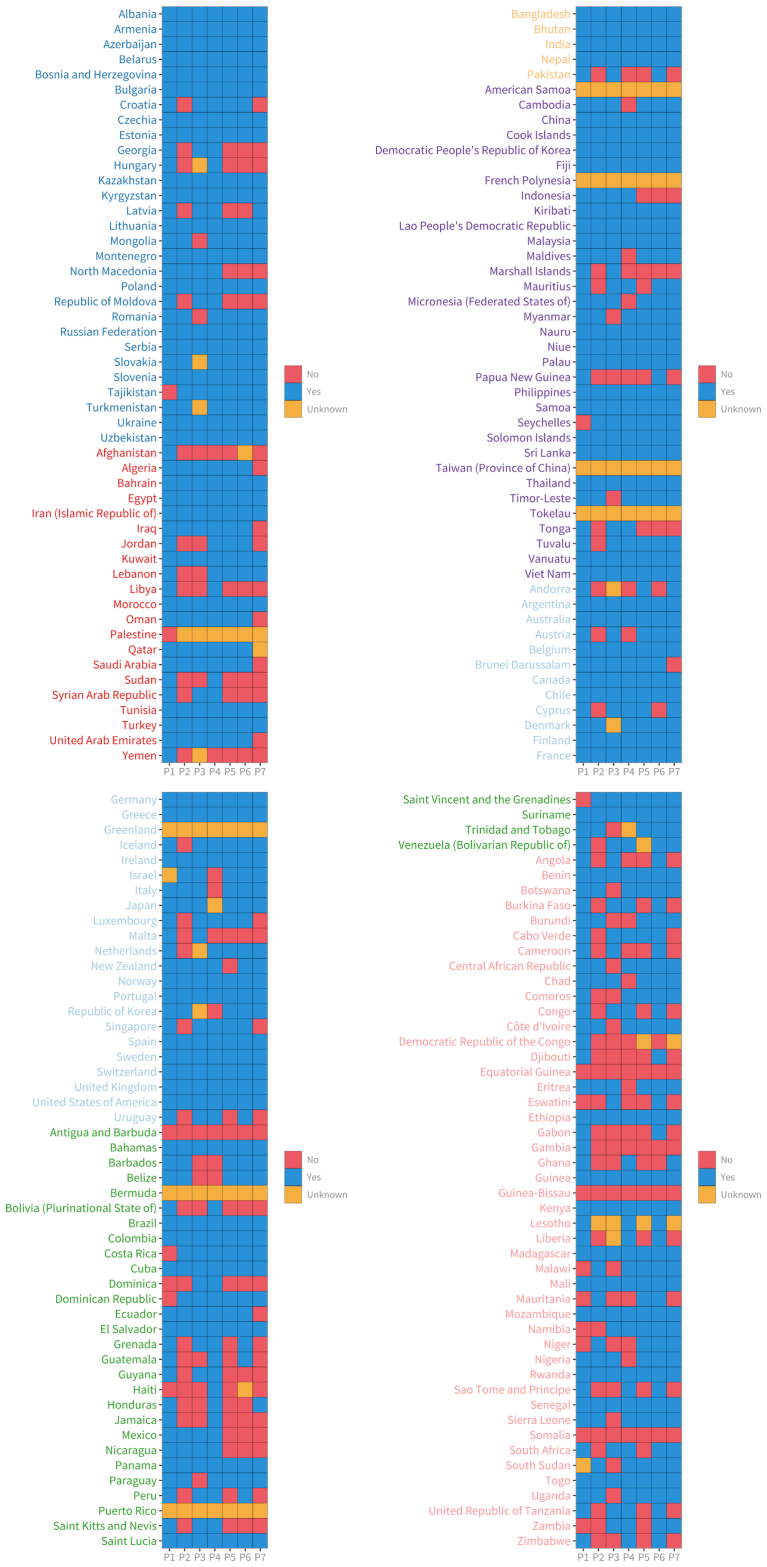
Policy implementation per country. *Note*: P1 – National tobacco control programmes; P2 – Action plan for CVDs; P3 – Operational Unit in Ministry of Health with responsibility for NCDs; P4 – Guidelines for the management of CVDs; P5 – Action plan to reduce physical inactivity; P6 – Action plan to reduce unhealthy diet related to NCDs; P7 – Action plan to reduce the harmful use of alcohol; P8 – Availability of CVD drugs (e.g., ACE inhibitors, aspirin, and Beta blockers) in the public health sector. Sources: Global Health Observatory. Available from: https://www.who.int/data/gho.

In the Sub-Saharan Africa region, over 50% of countries do not have a comprehensive CVD plan, an NCD Unit within the Ministry of Health, or general availability of CVD drugs in the public sector. North Africa and the Middle East had the lowest implementation rate for action plans to reduce the harmful use of alcohol. The Latin America and the Caribbean region had the lowest implementation rate for action plans to reduce physical inactivity and unhealthy diets related to NCDs (Figure 10).

## Discussion

In this paper we summarise the burden of CVDs, associated risk factors, the association between national health expenditures and CVD burden, and the role of critical policies, as presented in the World Heart Report 2023.

Cardiovascular diseases have remained the leading cause of mortality for over three decades, accounting for approximately one-third of all global deaths, with 20.5 million deaths recorded in 2021 [1]. The decline in CVD death rates has been faster in high-income countries compared to low- and middle-income countries, where over 80% of CVD deaths currently occur. Globally, CVDs have consistently been the leading cause of deaths over the last three decades. The Central Europe, Eastern Europe, and Central Asia region continue to face the highest levels of CVD mortality globally. Results indicate that although CVD mortality levels are generally lower in women than in men, almost 30% of countries in the North Africa and Middle East region and the Sub-Saharan Africa region (mainly West African countries) report higher CVD mortality rates for females than males. The association between health expenditure as a percentage of GDP, out-of-pocket expenditure, and CVD mortality highlights the need for adequate investment in healthcare systems to mitigate the burden of CVD. In terms of risk factors, raised blood pressure is the leading global CVD risk factor, contributing to approximately 10 million deaths in 2019. Most CVD risk factors, including physical inactivity, alcohol consumption, tobacco smoking, raised blood pressure, and diabetes, are more prevalent in men compared to women, with obesity being the only risk factor higher in women.

While the data presented here provide a detailed picture of the current burden of CVDs, there are some important limitations. The data used to present the temporal burden of CVDs and associated risk factors are derived from complex modelling [10,11]. Richness/lack of data informing the model is statistically expressed through the levels of uncertainty. Here, for simplicity, we have not systematically reported the associated levels of uncertainty. In particular, the lack of data from low- and middle-income countries, results in larger levels of uncertainty in those countries.

There is no one-size-fits-all approach to improving cardiovascular health globally. Every population is susceptible to different risk factors based on where they live and their lifestyles. Whether that is having higher prevalence of tobacco and alcohol use and higher sodium intake or being more exposed to dangerous levels of air pollution and having lower levels of physical activity. This means that decision makers and stakeholders must look closely at the risk factor prevalence in their countries and regions to fully understand what policy areas need more focus to get cardiovascular health moving in the right direction. There are, however, baseline approaches that every country should implement as a foundation from which to build tailored activities to tackle CVDs. This includes implementing major policy initiatives that are essential to improving CVD health, such as National Tobacco Control Programmes, securing the availability of CVD drugs, and creating an Operational Unit in the Ministry of Health responsible for tackling NCDs. Additionally, it requires adequately funded health systems and initiatives so that all communities can access the care they need. The stalling progress in cardiovascular health is not unique. Almost every health initiative around the world suffered because of the COVID-19 pandemic and countries are now grappling with which areas to prioritise as they aim to boost and protect the health of their populations. Given the severe burden of CVDs both in terms of mortality and morbidity, this area of health cannot be neglected. The world will struggle to meet the ambitious targets set to reduce premature mortality from NCDs by 25% compared to 2010 levels, by 2025. There is still time, however, to accelerate action toward meeting Sustainable Development Goal 3.4 of reducing by one-third premature mortality from NCDs, including cardiovascular diseases.

To promote action against CVDs, several aspects should be considered in the short and long term. First, countries need to enhance data collection on CVDs and their risk factors, particularly in low- and middle-income countries where data gaps exist, to better understand populations at higher risk. Second, countries should aim for health expenditure of at least 5% of GDP, in line with WHO recommendations. Third, countries should implement evidence-based policies to address CVDs, considering the disease burden and prevalent risk factors, while ensuring adequate resources and monitoring for progress. Fourth, it is crucial to prioritise coverage of CVD prevention and management interventions within Universal Health Coverage (UHC) benefit packages to minimise out-of-pocket expenses. Fifth, lessons learned from successful CVD prevention, management, and improved access to care and therapies should be implemented across all regions to address inequities and uneven progress in reducing CVD mortality.

The WHF Policy Index described here provides a working tool for national governments to use to assess, monitor and improve implementation of policies addressing CVDs health. We must recognise that policies are only as strong as when they are enforced and evaluated. Here we attempt to begin capturing measures by which organisations and advocates can monitor the actions of key stakeholders like the government and other players at the national level.

It is imperative to take concerted action to combat cardiovascular diseases and work towards achieving the global targets set forth, thereby improving the health outcomes and well-being of populations worldwide.

## Footnotes

The World Heart Report, launched in May 2023, is aimed at equipping policymakers and advocates around the world with the information needed to help reduce CVD deaths and accelerate progress in cardiovascular health. The report findings highlight the main differences between geographies in terms of CVD burden and risk factors, as well as structural barriers and inequities in CVD health, with the goal of guiding policymakers at national and international levels toward the priorities they should seek to address. It is available from https://world-heart-federation.org/resource/world-heart-report-2023.

Premature mortality from non-communicable diseases refers to deaths occurring between the ages of 30 and 70 years. These premature deaths not only result in a significant loss of life for individuals but also have broader impacts on societies and economies, particularly as they affect individuals within the working age range.
